# Monophasic synovial sarcoma presenting as a primary ileal mass: a case report and review of the literature

**DOI:** 10.1186/1752-1947-6-83

**Published:** 2012-03-13

**Authors:** Alaa N Alsharief, Musa Fageeh, Yousof Alabdulkarim

**Affiliations:** 1 King Fahad Medical City, Riyadh, Saudi Arabia, P.O. Box 59046, Riyadh 11525 Kingdom of Saudi Arabia

## Abstract

**Introduction:**

Synovial sarcoma is a rare malignant mesenchymal tumor mainly arising in the peri-articular tissue in young adults. There are few cases reported in other areas.

**Case presentation:**

We report the case of a 29-year-old Saudi woman of Arabian ethnicity with synovial sarcoma arising primarily from the ileum who presented with abdominal pain, a palpable mass and incomplete intestinal obstruction. A literature review was performed to gather information on this rare gastrointestinal tract sarcoma.

**Conclusions:**

Although it is a rare tumor of the pre-articular tissues, synovial sarcoma can present, in exceedingly rare cases, in unusual anatomical sites such as the gastrointestinal tract. We believe the reporting of all rare or unexpected presentations of sarcoma will eventually improve our understanding of this relatively unusual malignancy.

## Introduction

Synovial sarcoma is a malignant mesenchymal tumor of uncertain histogenesis. It may be biphasic, monophasic, or poorly differentiated [1]. Synovial sarcoma is a rare entity representing 5% to 10% of all soft tissue sarcomas, typically occurring around the joints, mainly the knee [2,3]. Occasionally, it arises in the head and neck [4,5], lungs [6-8], heart [9], retroperitoneum [10], prostate [11] and intra-neurally [12]. There are few cases reported of synovial sarcoma arising in the gastrointestinal tract. We report the case of primary synovial sarcoma arising in the ileum of a 29-year-old woman. Our patient's tumor displayed morphologic features and immunohistochemical staining consistent with this disease. We believe that our case is the first reported case of monophasic synovial sarcoma arising from the ileum.

## Case presentation

A 29-year-old Saudi woman presented to our Emergency Room with a five-day history of epigastric and lower abdominal pain associated with lower abdominal and pelvic fullness that she attributed to fibroids. The pain was intermittent, colicky in nature and associated with nausea and vomiting. She denied any history of fever and there had been no change in her bowel habits or urinary symptoms. For several months she had been followed up regularly at the gynecology clinic due to the lower abdominal heaviness and distension. Her gynecological history revealed a nulliparous woman with no vaginal bleeding. Her medical history was significant for epilepsy since childhood, which was well controlled by medication, and a laparoscopic appendectomy one year ago. On examination, she was alert and conscious. Her vital signs were stable. Her abdomen was distended with a palpable pelvic-abdominal mass that was hard, non-tender and slightly mobile. Her liver and spleen were within the normal range and she had normal bowel sounds. No lymph nodes were enlarged and digital rectal examination revealed no abnormalities.

**Figure 1:**
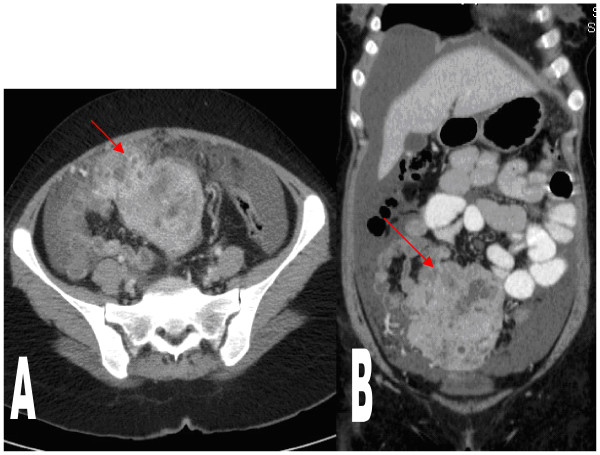
**(A, B) Computed tomography (CT) axial and coronal cuts showing the pelvic/abdominal heterogeneous mass with areas of necrosis in addition to ascites**.

A computed tomography (CT) scan of her abdomen and pelvis showed a pelvic/abdominal mass adjacent to the distal small bowel loops separate from the uterus and ovaries, heterogeneously enhancing, measuring roughly 65 × 99 mm with multiple areas of necrosis (Figure 1A, B). A CT scan of her chest showed indeterminate multiple bilateral subpleural 2 mm to 4 mm lung nodules, too small to biopsy.

The case was discussed by the multidisciplinary tumor board and the advice was to confirm the diagnosis to eliminate the possibility of an ovarian origin of the tumor because of its very close proximity.

Surgical exploration was indicated due to the finding of a large mass that was composed of a cystic and also a large solid component that needed to be investigated to eliminate the possibility of lymphoma or tuberculosis, which is quite common in our population. However, there was some radiological confusion about the origin of this mass and to some colleagues it was necessary to confirm it with a histological biopsy. For this reason, our patient underwent diagnostic laparoscopy, which showed a moderate amount of ascetic fluid with a large mass in the lower abdomen. When a biopsy was attempted, a large vein on the mass ruptured and bleeding made visualization extremely difficult. Therefore, the procedure was converted to an open one through a small lower midline incision. A 10 × 10 cm mass arising from the terminal ileum, occupying the pelvis and reaching to the umbilical area was found.

The uterus, ovaries and the urinary bladder were not involved. After a minimal dissection the tumor was excised with 10 cm free margins of small bowel and primary side-to-side anastomosis was performed. Our patient tolerated the procedure very well and her recovery period was unremarkable. She was discharged home on the seventh post-operative day. She was reviewed on an out-patient basis by our colleagues in radio-oncology and due to the radical nature of the surgery she was not offered any adjuvant treatment. She is still being followed up by our surgical oncology team and has shown no evidence of local or distant metastasis based on clinical and CT scan results after 24 months of follow-up.

**Figure 2:**
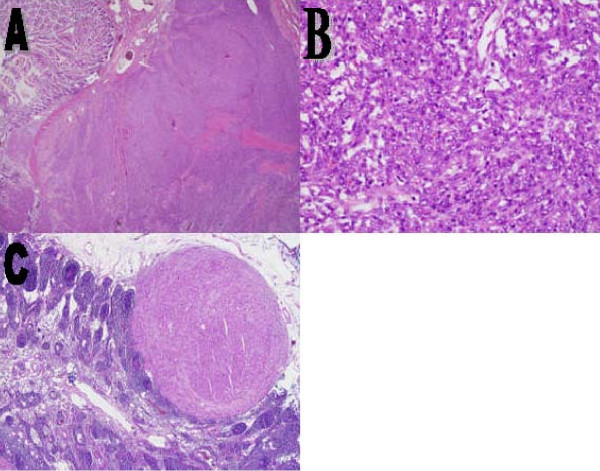
**(A) A spindle cell neoplasm arising from the wall of the small bowel and pushing the mucosa toward the lumen, (B) spindle cells with mild to moderate degree of pleomorphism and few conspicuous nuclei, and (C) a spindle cell metastatic focus in a mesenteric lymph node (hematoxylin and eosin; (A) ×2, (B) ×20, (C) ×4)**.

Gross pathology revealed a segment of small bowel measuring 17 × 2 cm. It was adherent to a portion of ascending colon. An 8 × 7 × 3 cm white solid mass with a homogeneous cut surface was bulging from the serosal side of the small bowel; it was 4 cm away from the proximal resection margin. The tumor was adherent to the large bowel but not infiltrating it grossly. Few lymph nodes were identified within the mesenteric fat. Microscopy showed that the tumor was composed of cellular spindle cells with a mild to moderate degree of pleomorphism arising from the small bowel wall and mainly involving the submucosa, muscularis propria and serosa. Focal mucosal erosion was identified. The tumor cells showed a moderate amount of eosinophilic cytoplasm, evenly distributed nuclear chromatin and a few conspicuous nuclei (Figure 2A, B). The mitotic count was one to two per 10 high-power fields. A metastatic focus was identified within a single mesenteric lymph node (Figure 2C).

**Figure 3:**
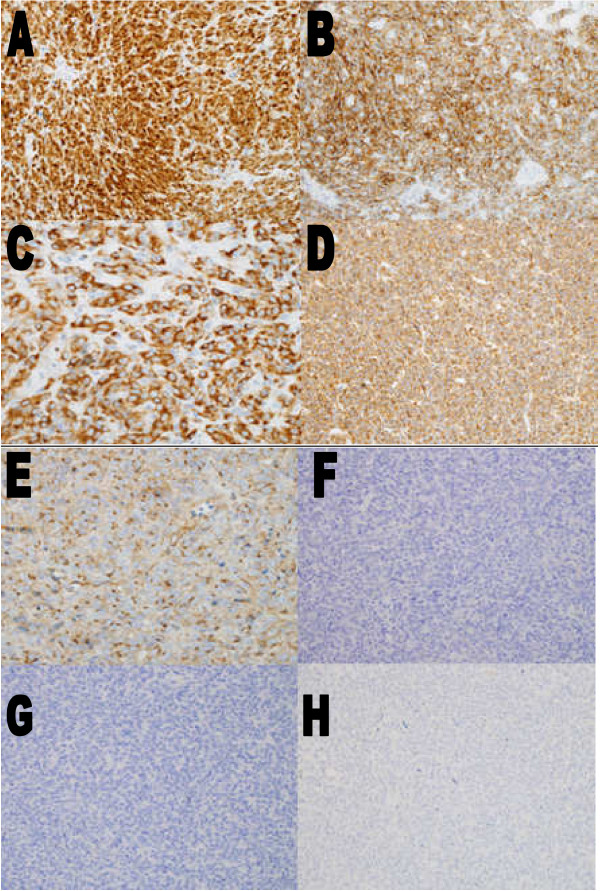
**Immunohistochemical staining. (A) **S100, ×20; **(B) **epithelial membrane antigen (EMA), ×20; **(C) **B cell lymphoma 2 (Bcl 2), ×40; **(D) **vimentin, ×20; **(E) **CD117, ×20; **(F) **CD99, ×20; **(G) **desmin, ×20; **(H) **cytokeratin (CK)5/6, ×20.

Immunohistochemical testing results showed that the tumor was positive for S100, epithelial membrane antigen (EMA), B cell lymphoma 2 (Bcl 2), and vimentin (Figure 3A-C). CD99 tests were negative (Figure 3F) and CDl17 tests were positive (Figure 3E), in addition to calretinin (focal) and synaptophysin (focal), and CD56 (few cells). The specimen tested negative for CD34, CAM5.2, cytokeratin 7, 20, and 5/6 (Figure 3H), cytokeratin AE1/AE3, neuron-specific enolase (NSE), CD31, desmin (Figure 3G), chromogranin, smooth muscle actin (SMA), HMB45, factor 8, Ber/EB4, and melan-A.

A cytogenetic study of the specimen was conducted on a formalin fixed paraffin embedded tumor sample. The result of a real-time polymerase chain reaction (RT-PCR) procedure for synovial sarcoma-associated fusion transcript (SYT-SSX1 and SYT-SSX2) was negative.

Our patient was seen periodically in our surgical oncology clinic and she has remained disease free for 24 months with no clinical or radiological evidence of local recurrence.

## Discussion

Gastrointestinal sarcoma accounts for 0.1% to 3% of all gastrointestinal (GI) malignancies and approximately 10% of all sarcomas [13,14]. In the ileum, the majority of malignant sarcomas are GI stromal tumors (GISTs) [15]. To the best of our knowledge there have been only 27 reported cases of synovial sarcoma arising in the GI tract, and none of them in the ileum [16-32]. All of them shared the diagnostic histology and immunohistochemical features of synovial sarcoma. In 64% of the cases, the characteristic (X; 18) translocation was identified. We made the diagnosis in our patient's case based on the histology of monophasic synovial sarcoma and the Immunohistochemical features. Although the molecular assay results were negative for SYT-SSX1 and SYT-SSX2, the combined histological and Immunohistochemical profiles were highly characteristic of monophasic synovial sarcoma.

The median age at diagnosis was 41.5 years (range, 14 to 75 years). There is no significant sex predilection with a male to female ratio of 1:1.15. There are 11 and 12 cases of GI tract synovial sarcoma arising from the esophagus (including one from the gastroesophageal junction) and stomach, respectively, representing the most common sites. The presenting symptoms were pain, obstruction or bleeding. The histological subtypes were monophasic in 15 patients and biphasic in 11 patients including two patients with a poorly differentiated component. The remaining patients' histological subtypes have not been reported. All of the reported cases were treated surgically. The number of patients who received adjuvant chemotherapy or radiotherapy or both are five, four and three, respectively. Only one patient had metastasis at first presentation. Billings [25] reported a patient with gastric synovial sarcoma with multiple liver metastases who died of the disease after six months. The survival period after diagnosis of all reported cases ranged from 1 to 224 months (Table 1).

**Table 1:** Summary of the reported cases of primary synovial sarcoma in the gastrointestinal tract

Author, year, reference	Location	Presenting symptoms	Age, years	Gender	Size, cm	Gross features	Histologic type	Translocation	Treatment	Status and follow-up, months
Palmer *et al. *1983 [16]	Esophagus	Dysphagia	75	F	2.5	Polypoid	Biphasic	-	S+Rad	DOD, 24
Amr *et al. *1984 [17]	Esophagus	Dysphagia	25	M	5	Polypoid	Biphasic	-	S+Rad	AWOD, 36
Bloch *et al.*, 1987 [18]	Esophagus	Dysphagia, dyspnea	15	M	7	Polypoid	Biphasic	-	S+Rad	AWOD, 36
Pulpiero *et al.*, 1988 [19]	Esophagus		24	M			Biphasic	-	S	NR
Caldwell *et al.*, 1991 [20]	Esophagus		29	F			NR	-	S+Rad+Chemo	AWOD, 195
Perch *et al.*, 1991 [21]	Esophagus		15	M			Biphasic	-	S+Rad	AWOD, 5 to 6 years after surgery
Antón-Pacheco *et al.*, 1996 [22]	Esophagus	Dysphagia, weight loss	14	F	7	Polypoid	Biphasic	-	S+Chemo+Rad	AWOD, 30
Habu S *et al. *1998 [23]	Esophagus	Sensation of something stuck in the throat	20	M	8	Polypoid	Biphasic	-	S+Chemo+Rad	AWOD, 20
Bonavina *et al.*, 1998 [24]	Esophagus	Achalasia	63	F		Polypoid	NR	-	-	-
Billings *et al.*, 2000 [25]	Gastroesophageal junction	Incidental finding for pyloric stenosis	47	M	5.2	Polypoid	Biphasic	t(X; 18)	S	AWOD, 21
Billings *et al.*, 2000 [25]	Stomach	Abdominal pain, nausea, vomiting and rectal bleeding	55	F	16	Spherical, intramural	Biphasic and PDSS	t(X; 18)	S	DOD, 6
Chan *et al.*, 2004 [26]	Jejunum	Epigastric pain, vomiting and fever	28	M	15	Polypoid, intramural	Monophasic	t(X; 18), SSX2	S	DOD, 1
Butori *et al.*, 2006 [27]	Esophagus	Dysphagia	72	F	11	Polypoid	Biphasic	t(X; 18)	S+Chemo	6
Akhunji *et al.*, 2007 [28]	Stomach	Epigastric pain	42	M	11		Biphasic	t(X; 18)	S+Chemo	DOD, 24
Parfitt *et al.*, 2007 [29]	Colon	Rectal bleeding	32	M	2	Polypoid	Monophasic	t(X; 18)	S	5
Schreiber-Fracklam *et al.*, 2007 [30]	Distal duodenum	Abdominal pain	39	F	5	Polypoid	Monophasic	t(X; 18), SSX2	S+Chemo	Recurrence 8 months after surgery
Makhlouf *et al.*, 2008 [32]	Stomach		67	F	0.8		Monophasic	t(X; 18)	S	AWOD, 12
Makhlouf *et al.*, 2008 [32]	Stomach		49	M	2		Monophasic with a poorly differentiated component	t(X; 18)	S	DOD, omental metastasis, 29
Makhlouf *et al.*, 2008 [32]	Stomach		68	F	2		Monophasic	t(X; 18)	S	AWOD, 22
Makhlouf *et al.*, 2008 [32]	Stomach		29	M	2.8		Monophasic	t(X; 18)	S	AWOD, 224
Makhlouf *et al.*, 2008 [32]	Stomach, gastrodudenal junction		54	F	3		Monophasic	t(X; 18)	S	Recent case
Makhlouf *et al.*, 2008 [32]	Stomach		58	F	3		Monophasic	t(X; 18)	S	AWOD, 21
Makhlouf *et al.*, 2008 [32]	Stomach		37	F	4		Monophasic	t(X; 18)	S	Local recurrence, re-excised. DOC 48
Makhlouf *et al.*, 2008 [32]	Stomach		50	M	6		Monophasic	t(X; 18)	S+Chemo	Alive with recurrence, 6
Makhlouf *et al.*, 2008 [32]	Stomach		42	M	8	Polypoid	Biphasic	t(X; 18)	S+Chemo	DOD, 25
Makhlouf *et al.*, 2008 [32]	Stomach		66	F	15	Polypoid	Monophasic	t(X; 18)	S	Lost to follow-up
Company *et al.*, 2009 [31]	Proximal duodenum	Weight loss asthenia anorexia, nausea, epigastric pain	69	F	8	Spherical, intramural	Monophasic	t(X; 18)	S	Died due to complications, 1
Present case, 2010	Ileum	Abdominal pain, distension and heaviness	39	F	8	Intramural	Monophasic	-	S	AWOD, 6

The second case reported by Billings [25] could represent a metastatic focus of a primary neck tumor as it showed the same histopathological features.

AWOD = alive without evidence of disease; AWD = alive with residual disease; Chemo = chemotherapy; DOC = died of other cause; DOD = died of disease; NR = not reported; Rad = radiotherapy; S = surgery; SSX = synovial sarcoma-associated fusion transc; PDSS = Poorly difrentiated synovial sarcoma

In soft tissue synovial sarcoma, numerous studies over the years have reported that it is a high grade malignancy with a high rate of metastasis leading to death; 10-year survival rates between 24% to 68% and 11% to 56% have been reported [27,33].

In the article by Bergh *et al. *[33], the investigators divided patients with synovial sarcoma into low-risk and high-risk groups depending on their age, tumor size and grade as follows: low-risk group (patient age < 25 years, tumor size < 5 cm, and no histologic evidence of poorly differentiated tumor); high-risk group (patient age approximately 25 years, tumor size approximately 5 cm, and poorly differentiated tumor). The question is, can we apply the same risk factors to patients with GI synovial sarcoma? To answer such a question we need to diagnose more cases and collect more data about the already published cases to study the behavior of this disease entity in the GI tract, as well as in other parts of the body. Presently, the evidence suggests adequate primary surgery is essential to both local control and outcome. The other question that one may ask: why do these tumors shift to unusual sites and different tissues? The honest answer, at least at this time, would by that we do not yet know the exact mechanism for such a shift. We are involved in a larger research study to investigate the increased incidence of sarcomas in the Saudi population, particularly in the northern region, which is close to the military activities of both Gulf wars and the military activities in Iraq over the last decade.

## Conclusions

In general, sarcomas are relatively rare tumor entities, particularly in the GI tract. For many reasons, it is easy to confuse the subtypes of sarcomas, including the histological similarities, the experience of the centers dealing with such tumors, proper handling, and staining and immunohistochemical findings. It is also likely to mistake such extremely rare presentations of an unusual malignancy such as synovial sarcoma for relatively more common tumors such as GISTs, which can present with more or less the same clinical scenario. The clinical importance comes from the fact that sarcomas carry a slightly worse prognosis and need a closer follow-up not to miss any sign of local recurrence or distant metastasis. We also believe that any rare presentation of any subtype of sarcoma should be carefully reported and documented to add to the relatively small pool of cases in comparison with other common malignant tumors, in order to increase our knowledge and understanding of sarcoma in general.

## Consent

Written informed consent was obtained from the patient for publication of this case report and any accompanying images. A copy of the written consent is available for review by the Editor-in-Chief of this journal.

## Competing interests

The authors declare that they have no competing interests.

## Authors' contributions

AA collected the data and informed consent, wrote the manuscript, organized the text and photos and conducted the literature review. MF reviewed the histopathological diagnosis, provided the magnified histological slide photos, followed up with other universities for confirmation of histology and histochemical diagnosis. YA provided the surgical details, followed up our patient's progress and details of her history, and reviewed the article and revised the manuscript. All authors read and approved the final manuscript.
